# Drug Design and Discovery: Principles and Applications

**DOI:** 10.3390/molecules22020279

**Published:** 2017-02-13

**Authors:** Shu-Feng Zhou, Wei-Zhu Zhong

**Affiliations:** 1 Department of Bioengineering and Biotechnology, College of Chemical Engineering, Huaqiao University, Xiamen 361021, Fujian, China; 2 Gordon Life Science Institute, Belmont, MA 02478, USA

Drug discovery is the process through which potential new therapeutic entities are identified, using a combination of computational, experimental, translational, and clinical models (see, e.g., [1,2]). Despite advances in biotechnology and understanding of biological systems, drug discovery is still a lengthy, costly, difficult, and inefficient process with a high attrition rate of new therapeutic discovery. Drug design is the inventive process of finding new medications based on the knowledge of a biological target. In the most basic sense, drug design involves the design of molecules that are complementary in shape and charge to the molecular target with which they interact and bind. Drug design frequently but not necessarily relies on computer modeling techniques and bioinformatics approaches in the big data era. In addition to small molecules, biopharmaceuticals and especially therapeutic antibodies are an increasingly important class of drugs and computational methods for improving the affinity, selectivity, and stability of these protein-based therapeutics have also gained great advances [3]. Drug development and discovery includes preclinical research on cell-based and animal models and clinical trials on humans, and finally move forward to the step of obtaining regulatory approval in order to market the drug. Modern drug discovery involves the identification of screening hits, medicinal chemistry and optimization of those hits to increase the affinity, selectivity (to reduce the potential of side effects), efficacy/potency, metabolic stability (to increase the half-life), and oral bioavailability. Once a compound that fulfills all of these requirements has been identified, it will begin the process of drug development prior to clinical trials. 

This Special Issue “Drug Design and Discovery: Principles and Applications” was focused on the basic principles of modern drug design and discovery and the potential applications. It covered seventeen research articles and one communication contributed from experts all around the world, as briefed below.

The 2014 Ebola epidemic in West Africa is believed to have caused more than 11,000 fatalities. The request for novel drug development, finding efficient drug discovery pathways is going to be crucial in the fight against future outbreaks. In the article entitled “Combating Ebola with Repurposed Therapeutic Using the CANDO Platform” [4], Gaurav Chopra, Ram Samudrala, and coauthors have developed a Computational Analysis of Novel Drug Opportunities (CANDO) platform based on the hypothesis that drugs function by interacting with multiple protein targets to create a molecular interaction signature that can be exploited for rapid therapeutic repurposing and discovery. They used the CANDO platform to generate top ranking drug candidates for Ebola virus disease treatment, which were compared to those identified from in vitro studies. They found that integrating computational docking predictions on a proteomic scale with results from in vitro screening studies may be used to select and prioritize compounds for further in vivo and clinical testing. This approach will significantly reduce the lead time, risk, cost, and resources required to determine efficacious therapies against future Ebola virus disease outbreaks.

Wei Xiao, Huiming Hua, Jinyi Xu, and their coworkers wrote an article with the title “NO-Releasing Enmein-Type Diterpenoid Derivatives with Selective Antiproliferative Activity and Effects on Apoptosis-Related Proteins” [5]. They designed and synthesized a series of nine enmein-type ent-kaurane diterpenoid and furoxan-based nitric oxide (NO) donor hybrids from commercially available oridonin. Their investigation in antiproliferative activity of these hybrids suggested that these kinds of NO-donor/diterpenoid hybrids could provide a promising approach for the discovery of novel antitumor agents.

Dimitra Hadjipavlou-Litina and colleagues presented an exhaustive docking analysis considering the case of autotaxin in the article entitled “Boronic Acid Group: A Cumbersome False Negative Case in the Process of Drug Design” [6]. They found that virtual screening of large libraries of boronic acid derivatives fail to dock in a natural mode. They are left out as false negatives both in regards to their binding poses and their scoring function values. To solve the problems encountered, the authors characterized the formed bond between Ser/Thr residues more accurately as a polar covalent bond instead of as a simple nonpolar covalent bond based on natural bond orbital calculations. The findings described in this article highlight general options that need to be considered when large libraries of boron compounds are virtually screened to identify novel hits in drug design. 

In their article “Antiproliferative Activity and Cellular Uptake of Evodiamine and Rutaecarpine Based on 3D Tumor Models” [7], Feng Xu and coauthors employed the 3D culture of MCF-7 and SMMC-7721 cells based on the hanging drop method and evaluated the anti-proliferative activity and cellular uptake of two promising anti-tumor drug candidates, evodiamine (EVO) and rutaecarpine (RUT), in 3D multicellular spheroids and compared the results with those obtained from 2D monolayers. They believe that their study provided a new vision of the anti-tumor activity of EVO and RUT via 3D multicellular spheroids and cellular uptake through the fluorescence of compounds and may be helpful for drug screening and cytotoxicity studies. 

Malaria is one of the principal diseases of developing countries, particularly in Africa, Asia, and South America. Due to the toxic side effects and the risk of developing resistance after prolonged treatment with aminoquinolines, it demands a continuous effort to develop new antimalarial agents, especially as an effective vaccine for malaria is not available. Rizk E. Khidre and colleagues designed and synthesized a novel series of quinoline compounds and screened for their antimalarial activities, with the hope that these compounds could lead to the availability of better drugs to treat malaria. Their study results are presented in the article “New Potential Antimalarial Agents: Design, Synthesis and Biological Evaluation of Some Novel Quinoline Derivatives as Antimalarial Agents” [8].

As described in the article entitled “Novel (*E*)-β-Farnesene Analogues Containing 2-Nitroiminohexahydro-1,3,5-triazine: Synthesis and Biological Activity Evaluation” [9], Xinling Yang and coauthors introduced a series of novel (*E*)-β-farnesene analogues by replacing the conjugated double bonds of EβF with 2-nitroiminohexahydro-1,3,5-triazine. Their bioassay results showed that all the analogues displayed different repellent and aphicidal activities against the green peach aphid (Myzus persicae). They also performed a preliminary structure-activity relationship (SAR), which offered valuable clues for the design of new EβF analogues. 

In the search for prodrug analogs of clopidogrel with improved metabolic characteristics and antiplatelet bioactivity, a group of clopidogrel and vicagrel analogs selectively deuterated at the benzylic methyl ester group were synthesized, characterized, and evaluated by Yan Yang, Jingkai Gu, and their colleagues. The ability of the compounds to inhibit ADP-induced platelet aggregation and pharmacokinetics from rats after oral dosing were studied and the results are detailed in the article “Significant Improvement of Metabolic Characteristics and Bioactivities of Clopidogrel and Analogs by Selective Deuteration” [10].

Interest in intranasal administration as a non-invasive route for drug delivery continues to grow rapidly. Because of the sensitive respiratory mucosa, not only the active ingredients, but also additives need to be tested in appropriate models for toxicity. Rita Ambrus and coworkers studied the cytotoxicity of six pharmaceutical excipients, which could help to reach larger residence time, better permeability, and an increased solubility dissolution rate. As described in the communication entitled “Cytotoxicity of Different Excipients on RPMI 2650 Human Nasal Epithelial Cells” [11], they found that all additives at 0.3% sodium hyaluronate and polyvinyl alcohol at 1% concentrations can be safely used for nasal formulations. 

As spermatozoa become mature and acquire fertilizing capacity during their passage through the epididymal lumen, Li-Juan Qu, Yan Zhu, et al. conducted a study to identify new epididymal luminal fluid proteins involved in sperm maturation in infertile rats by dutasteride, a dual 5α-reductase inhibitor, in order to provide potential epididymal targets for new contraceptives and infertility treatments. They report for the first time that dutasteride influences the protein expression profiling in the epididymal luminal fluids of rats, and this result provides some new epididymal targets for male contraception and infertility therapy. The study results are presented in the article “Identification of New Epididymal Luminal Fluid Proteins Involved in Sperm Maturation in Infertile Rats Treated by Dutasteride Using iTRAQ” [12].

Reported in the article “Synthesis and Evaluation of Ester Derivatives of 10-Hydroxycanthin-6-one as Potential Antimicrobial Agents” [13], Jun-Ru Wang and coauthors studied a new series of ester derivatives of 10-hydroxycanthin-6-one using a simple and effective synthetic route as part of their continuing research on canthin-6-one antimicrobial agents. They characterized the structure and antimicrobial activity of each compound, investigated the structure-activity relationship, and identified the promising lead compound that had significant antimicrobial activity against all the fungi and bacterial strains tested for the development of novel canthine-6-one antimicrobial agents. 

Chun-Mei Jin, Zhe-Shan Quan, and colleagues wrote an article entitled “Synthesis and Biological Evaluation of Novel Benzothiazole Derivatives as Potential Anticonvulsant Agents” [14]. Because of the crucial need to develop more effective antiepileptic drugs endowed with an improved safety profile, the authors investigated new benzotriazoles with a mercapto-triazole and other heterocycle substituents, and evaluated their anticonvulsant activity and neurotoxicity for each compound by using the maximal electroshock, subcutaneous pentylenetetrazole, and rotarod neurotoxicity tests. The study outcomes are presented in their paper [14].

Non-steroidal anti-inflammatory drugs are the most commonly prescribed anti-inflammatory and pain relief medications. However, their use is associated with many drawbacks. In an attempt to circumvent these risks, Ahmed M. Gouda and coworkers designed, synthesized, and evaluated a set of N-(4-bromophenyl)-7-cyano-6-substituted-H-pyrrolizine-5-carboxamide derivatives as dual COX/5-LOX inhibitors. In light of their findings, these novel pyrrolizine-5-carboxamide derivatives represent a promising scaffold for further development into potential dual COX/5-LOX inhibitors with safer gastric profiles. Their results are detailed in the article “Design, Synthesis, and Biological Evaluation of Some Novel Pyrrolizine Derivatives as COX Inhibitors with Anti Inflammatory/Analgesic Activities and Low Ulcerogenic Liability” [15].

The main step in a successful drug discovery pipeline is the identification of small potent compounds that selectively bind to the target of interest with high affinity. In the work reported in the article “Self Organizing Map-Based Classification of Cathepsin k and S Inhibitors with Different Selectivity Profiles Using Different Structural Molecular Fingerprints: Design and Application for Discovery of Novel Hits” [16], Hany E. A. Ahmed and colleagues proposed an affordable machine learning method to perform compound selectivity classification and prediction. They collected compounds with reported activity and built a selectivity database formed of 153 cathepsin K and S inhibitors that are considered of medicinal interest. The study results exhibited the capability of the method in the design of further novel inhibitors with high activity and target selectivity.

Vancomycin, a widely used antibiotic, often induces nephrotoxicity; however, the molecular targets and underlying mechanisms of this side effect remain unclear. In order to uncover the comprehensive and global understanding on the effect of vancomycin, Zhi-Ling Li and Shu-Feng Zhou investigated the molecular targets of vancomycin in human proximal tubule epithelial HK-2 cells with a focus on cell cycle, apoptosis, autophagy, and epithelial to mesenchymal transition (EMT) pathways. The quantitative SILAC-based proteomic approach showed that vancomycin regulated cell proliferation, mitochondria-dependent apoptotic pathway and autophagy, and EMT in HK-2 cells, involving a number of key functional proteins and related molecular signaling pathways. This study may provide a clue to fully identify the molecular targets and elucidate the underlying mechanism of vancomycin-associated nephrotoxicity, resulting in an improved therapeutic effect and reduced side effect in clinical settings. Detailed results are presented in the article “A SILAC-Based Approach Elicits the Proteomic Responses to Vancomycin-Associated Nephrotoxicity in Human Proximal Tubule Epithelial HK-2 Cells” [17].

Knowledge of protein-protein interactions and their binding sites is indispensable for in-depth understanding of the networks in living cells. With the avalanche of protein sequences generated in the postgenomic age, it is critical to develop computational methods for identifying in a timely fashion the protein-protein binding sites (PPBSs) based on the sequence information alone because the information obtained by this method can be used for both biomedical research and drug development. To address such a challenge, Jianhua Jia, Bingxiang Liu, and colleagues [18] have proposed a new predictor, called iPPBS-Opt, in which they have used the concept of pseudo amino acid composition (PseAAC) [19] to formulate complicated protein sequences. Although there are many investigators (see, e.g., [20,21,22,23]) who also used the PseAAC to formulate protein sequences, this is the first time the stationary wavelet transform approach is introduced to reflect the functions of low-frequency phonons in proteins as deduced some 40 years ago [24,25]. Furthermore, to maximize the convenience for most experimental scientists, they have provided a step-by-step guide on how to use the predictor’s web server (http://www.jci-bioinfo.cn/iPPBS-Opt) to obtain the desired results without the need to go through the complicated mathematical equations involved. 

In the article “Synthesis of Canthardin Sulfanilamides and Their Acid Anhydride Analogues via a Ring-Opening Reaction of Activated Aziridines and Their Associated Pharmacological Effects” [26], Mei-Hsiang Lin and coworkers reported their investigation to find new cantharidinimides and related imides containing the sulfonamide group. The modification of cantharidinimide by means of the reaction of activated aziridine ring opening led to the discovery of a novel class of antitumor compounds. They found that the most potent cytostatic compound, *N*-cantharidinimido-sulfamethazine, exhibited anti-HL-60 and anti-Hep3B cell activities. Detailed results of their investigation are presented in the article [26].

Jian Li and coworkers wrote an article entitled “Chemical Structure-Related Drug-Like Criteria of Global Approved Drugs” [27]. They uncovered three important structure-related criteria closely related to drug-likeness, namely: (1) the best numbers of aromatic and non-aromatic rings are 2 and 1, respectively; (2) the best functional groups of candidate drugs are usually -OH, -COOR, and -COOH in turn, but not -CONHOH, -SH, -CHO, and -SO_3_H. In addition, the -F functional group is beneficial to CNS drugs, and the -NH_2_ functional group is beneficial to anti-infective drugs and anti-cancer drugs; (3) the best R value intervals of candidate drugs are in the range of 0.05–0.50 (preferably 0.10–0.35), and the R value of candidate CNS drugs should be as small as possible in this interval. They envision that the three chemical structure-related criteria may be applicable in a prospective manner for the identification of novel candidate drugs and will provide a theoretical foundation for designing new chemical entities with good drug-like properties.

For the purpose of finding highly active pyrazole amide compounds, Jin-Xia Mu, Xing-Hai Liu, Bao-Ju Li, and their coworkers designed and synthesized a series of novel pyrazole amide derivatives by multi-step reactions from phenylhydrazine and ethyl 3-oxobutanoate as starting materials. They characterized the structures and antifungal activities of the title compounds and used DFT calculations to study the structure-activity relationships. Their results indicated that some of the title compounds exhibited moderate antifungal activity, as shown in the article “Design, Synthesis, DFT Study and Antifungal Activity of Pyrazolecarboxamide Derivatives” [28]. 

The eighteen articles published in this thematic issue “Drug Design and Discovery: Principles and Applications” are highlighted in the areas of computer-aided drug discovery and development, drug design and synthesis approaches, in vitro and in vivo pharmacological and toxicological evaluations, etc. These articles not only provided important information, but also generated many useful tools for drug discovery and development. These works showed that the in vitro and in vivo experiments complemented with computation methods are continuously improving the effectiveness and efficiency of drug discovery processes to select lead candidates with more favorable pharmacological, pharmacokinetics, and toxicological profiles. 

It is our intent that publication of this Special Issue can stimulate new strategies in drug design and provide new tools, approaches, and technologies to facilitate the evaluation of new drug candidates, leading to the rapid and successful development of novel, effective, and safe medicines for treating diseases [29]. 
